# A Segmented Low-Order Bistable Stochastic Resonance Method for Fixed-Distance Target Detection in Millimeter-Wave Fuze Under Rainy Conditions

**DOI:** 10.3390/s25123801

**Published:** 2025-06-18

**Authors:** Bing Yang, Kaiwei Wu, Zhe Guo, Yanbin Liang, Shijun Hao, Zhonghua Huang

**Affiliations:** School of Mechatronical Engineering, Beijing Institute of Technology, Beijing 100081, China; 3120215164@bit.edu.cn (B.Y.); 3120205163@bit.edu.cn (K.W.); guozhe127@bit.edu.cn (Z.G.); 3120195127@bit.edu.cn (Y.L.); 3120215165@bit.edu.cn (S.H.)

**Keywords:** millimeter-wave fuze, stochastic resonance, anti-rainfall interference, target detection

## Abstract

Millimeter-wave (MMW) fuze signals experience significant degradation in rainy environments due to combined raindrop-induced attenuation and scattering effects, substantially reducing echo signal-to-noise ratio (SNR) and critically impacting ranging accuracy. To address these limitations while satisfying real-time processing requirements, this study proposes (1) a novel segmented low-order bistable stochastic resonance (SLOBSR) system based on piecewise polynomial potential functions and (2) a corresponding fixed-distance target detection algorithm incorporating signal pre-processing, particle swarm optimization (PSO)-based parameter optimization, and kurtosis threshold detection. Experimental results demonstrate the system’s effectiveness in achieving a 9.94 dB SNR enhancement for MMW fuze echoes under rainy conditions, enabling reliable target detection at SNRs as low as −15 dB. Comparative analysis confirms the SLOBSR method’s superior performance over conventional approaches in terms of both SNR enhancement and computational efficiency. The proposed method significantly enhances the anti-rainfall interference capability of the MMW fuze.

## 1. Introduction

Fixed-distance fuzes are electronic devices that utilize radio frequency, laser, ultra-wideband, or millimeter-wave (MMW) technologies to trigger signals at preset distances [1]. Among these options, MMW fuzes have been prioritized in modern real-time fixed-distance systems owing to their superior range accuracy, robust electromagnetic interference resistance, and miniaturized design [2]. Given the complexity and variability of operational environments for MMW fuzes, these systems must maintain reliable stability under diverse climatic conditions. Particularly because of the comparable scales between the MMW signal wavelengths and the diameter of the raindrops, the MMW fuze signals suffer significant attenuation and scattering in rainy environments [3,4]. This physical phenomenon may potentially limit the operational effectiveness of MMW fuzes under precipitation conditions.

Initial research efforts focused on characterizing MMW detector echo signals under rainy conditions to elucidate raindrop-induced effects. Ishimaru [5] analyzed the fundamental attenuation and scattering mechanisms occurring in rainy conditions. Zang et al. [6] theoretically demonstrated a 45% reduction in detection range for MMW radar systems during rainfall. Yang et al. [7] established that rainfall effects must be accounted for in MMW Doppler fuze signal processing, especially for low radar cross-section (RCS) targets. Subsequently, Hasirlioglu [8] developed a stratified rain field model, experimentally validating substantial noise floor elevation in radar returns—a finding corroborated by later studies [9,10,11]. Zhan et al. [12] further identified rainfall-induced ranging inaccuracies that may compromise detonation precision. These collective findings demonstrate how SNR degradation fundamentally impacts fuze reliability, highlighting the need for effective anti-rain interference solutions.

Substantial research efforts have explored various SNR enhancement methodologies. Wang et al. [13] utilized Empirical Mode Decomposition (EMD) to decompose radar echoes into intrinsic mode functions, suppressing environmental noise through autocorrelation energy analysis. Zhou et al. [14] applied Variational Mode Decomposition (VMD) to MMW fuze echoes, reconstructing target components via Pearson correlation coefficients, thereby enhancing anti-jamming performance. Pang et al. [15] developed a dual-VMD-correlation algorithm to isolate target signals from smoke interference in laser fuzes, while Lu et al. [16] proposed a variable-step adaptive filtering (AF) method for linear frequency modulation fuzes to achieve in-band noise suppression. Unfortunately, these methods exhibit inherent limitations: EMD-based methods suffer from mode mixing artifacts [17], VMD demonstrates sensitivity to parameter selection [17], and AF requires accurate estimation of reference signals [18]. Zhan et al. [12] proposed a convolutional neural network-based framework for rainfall interference suppression, which demonstrated high detection probability under rainy conditions. The effectiveness of this deep learning approach was found to be strongly dependent on the size of the training dataset [19]. After referring to alternative SNR enhancement approaches [20,21], this study attempts to employ the SR method to improve the SNR of echo signals under rainy conditions.

The SR method, originally conceptualized by Benzi in the 1980s [22], demonstrates a unique noise–energy transfer mechanism that enables significant SNR enhancement under specific conditions [23,24]. While classical bistable SR (CBSR) has been widely implemented, its effectiveness is fundamentally limited by output signal saturation phenomena [25]. To address this limitation, Qiao et al. [26] proposed the unsaturated bistable SR (UBSR) method by introducing first-order continuous potential barrier slopes via piecewise function implementation. This was followed by Chen et al.’s fractional exponential power bistable SR (FEPBSR) approach [27]. Both methodologies successfully overcome the saturation limitation while achieving theoretical SNR enhancement. These approaches have demonstrated exceptional performance in rolling bearing fault diagnosis applications. Further innovations include Liu et al.’s segmented asymmetric bistable SR system [28] incorporating asymmetry factors and Wang et al.’s asymmetric hybrid bistable SR (AHBSR) system [29] combining exponential and polynomial potential functions.

However, conventional SR implementations in MMW fuzes face two fundamental limitations: (1) the computational complexity induced by higher-order terms in potential functions and (2) restricted applicability to extremely low-frequency input signals [21]. To overcome these challenges, this study develops a novel segmented low-order bistable SR (SLOBSR) system. Its potential function combines second-/third-order terms in potential well areas with first-order terms at potential barrier boundaries. The SLOBSR method achieves dual optimization by: (1) enhancing signal-to-noise ratio (SNR) through noise-to-signal energy conversion under optimal parameter matching between target echoes, noise characteristics, and system configurations; (2) reducing computational complexity via low-order potential function implementation. Building upon this framework, a comprehensive fixed-distance target detection algorithm based on the SLOBSR system is developed, incorporating (i) input signal pre-processing, (ii) particle swarm optimization (PSO)-based parameter optimization, and (iii) kurtosis-based target detection. Both simulation and experimental results demonstrate that the proposed method achieves reliable target detection for MMW fuze echoes in rainy environments while maintaining computational efficiency.

The remainder of this paper is organized as follows: Section 2 systematically investigates the SNR degradation of MMW fuze echo signals under rainy conditions and identifies fundamental limitations in applying the conventional SR method to MMW ranging applications. Section 3 describes the fixed-distance target detection algorithm based on the SLOBSR system. Section 4 presents simulation results, followed by experimental validation in Section 5. Finally, Section 6 concludes the paper.

## 2. Problem Statement

### 2.1. SNR Reduction in MMW Fuze Under Rainy Conditions

**Figure 1:**
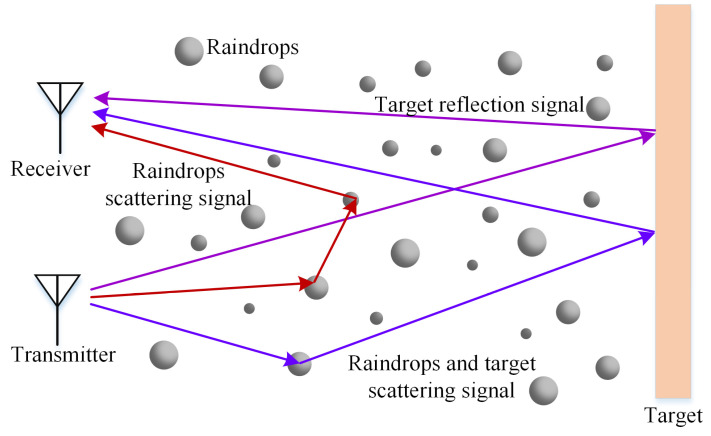
Detection of MMW fuze under rainy conditions.

Figure 1 demonstrates the detection process of MMW fuze signals under rainy conditions. The echo signals comprise three distinct components resulting from raindrop-induced scattering and attenuation effects: (1) target-reflected echo signals, (2) raindrop-scattered signals (incorporating both single and multiple scattering phenomena), and (3) composite signals jointly scattered by both raindrops and targets [5].

Consequently, the echo signal power can be expressed as follows:

(1) $$P_{r}=P_{rt}+P_{ra}+P_{rat},$$

where $P_{rt}$, $P_{ra}$, and $P_{rat}$ represent the power of echo signals from: (1) exclusive target reflection, (2) pure raindrop scattering, and (3) coupled target–raindrop scattering, respectively.

The target-reflected echo signal power can be determined using the fuze detection equation [6], expressed as follows:

(2) $$P_{rt}=\frac{P_{t}G^{2}\lambda^{2}\sigma_{t}}{{\left(4\pi \right)}^{3}R_{t}^{4}}e^{-2\gamma_{t}R_{t}}=P_{rt,no\_rain}e^{-2\gamma_{t}R_{t}},$$

where $P_{t}$ is the transmission signal power; G is transmitting and receiving antenna gain; λ is the signal wavelength; $\sigma_{t}$ is the RCS of targets; $R_{t}$ is the target distance; and $\gamma_{t}$ is the rainfall attenuation coefficient. $e^{-2\gamma_{t}R_{t}}$ denotes the attenuation coefficient of the target-reflected signal propagating through a rainfall environment [30], where this additional attenuation process is absent under no rain conditions ($\gamma_{t}=0$).

The raindrop-scattered echo signals comprise both single and multiple scattering components, with their power calculable as follows:

(3) $$P_{ra}=\sum_{n=1}^{\infty }\left(\frac{P_{t}G^{2}\lambda^{2}}{{\left(4\pi \right)}^{3}R_{s}^{2}}\prod_{i=1}^{n}\frac{\eta_{ai}}{R_{ai}^{2}}\right),$$

where $R_{s}$ is the propagation distance of MMW signals from the fuze transmitting antenna to the first intercepted raindrop; $R_{ai}$ is the propagation distance from one raindrop to the other raindrop or the receiving antenna; i is the scattering order; and $\eta_{ai}$ represents the energy loss of MMW signals due to raindrop scattering, expressed as follows:

(4) $$\eta_{ai}=\sigma_{si}F(\theta_{i})e^{-\gamma_{t}R_{ai}},$$

where $\sigma_{si}$ is the raindrop scattering cross-sectional area, and $F(\theta_{i})$ is the probability of the scattering phase function at the scattering angle $\theta_{i}$.

The echo signal power resulting from coupled raindrop–target scattering can be calculated as follows:

(5) $$P_{rat}=\sum_{n=1}^{\infty }\sum_{m=1}^{n}\left(\frac{P_{t}G^{2}\lambda^{2}}{{\left(4\pi \right)}^{3}R_{s}^{2}}\prod_{i=1}^{n}\frac{\eta_{ai}}{R_{ai}^{2}}\prod_{i=1}^{m}\frac{\sigma_{t}}{4\pi R_{ti}^{2}}\right),$$

where $R_{ti}$ is the propagation distance of MMW signals from the raindrop to the target.

Consequently, the SNR of MMW fuzes operating under rainy conditions is expressed as follows:

(6) $${SNR}_{rain}=10\log\left(\frac{P_{rt}}{P_{ra}+P_{rat}+PN}\right)=10\log\left(\frac{P_{rt,\;\;no\_rain}e^{-2\gamma_{t}R_{t}}}{P_{ra}+P_{rat}+PN}\right),$$

where PN is the receiver noise. Under no-rain conditions, the received signal consists solely of the target-reflected component, completely free from rainfall-induced attenuation effects and devoid of any rain-generated echo signals ($P_{ra}$ and $P_{rat}$) Therefore, the SNR of MMW fuzes operating under no-rain conditions can be expressed as follows:

(7) $${SNR}_{no\_rain}=10log(\frac{P_{rt,\;\;no\_rain}}{PN}),$$

Based on established SNR Formulas (6) and (7), the target echo signal experiences considerable attenuation during propagation through a rainfall environment while simultaneously accumulating rain-induced clutter noise. This dual mechanism of signal attenuation and noise amplification leads to pronounced SNR degradation under rainy conditions, thereby impairing reliable target detection and identification capabilities.

### 2.2. Limitations of Stochastic Resonance Systems

The SR system is characterized by cooperative interaction among signal, noise, and a nonlinear system. When the noise characteristics match the signal properties, the system facilitates noise-to-signal energy conversion, thereby enhancing the output signal SNR. This phenomenon can be described by the Langevin equation [31] as follows:

(8) $$\frac{dx}{dt}=-\frac{dU\left(x\right)}{dx}+S(t)+N(t),$$

where x is the system response; $U\left(x\right)$ is the system potential function; S(t) is the input periodic signal; and N(t) is the Gaussian white noise with zero mean and noise intensity D. The potential function of a CBSR is defined as follows:

(9) $$U(x)=-\frac{a}{2}x^{2}+\frac{b}{4}x^{4},$$

where potential function parameters a>0 and b>0. This equation is solved numerically by substituting Equation (9) into Equation (8) and implementing the fourth-order Runge–Kutta method [29] as follows:

(10) $$\left\{\begin{array}{l} k_{1}=-dU\left(x_{n}\right)/dx+S\left(n\right)+N(n)\\ k_{2}=-dU(x_{n}+hk_{1}/2)/dx+S(n)+N(n)\\ k_{3}=-dU(x_{n}+hk_{2}/2)/dx+S(n+1)+N(n+1)\\ k_{4}=-dU(x_{n}+hk_{3})/dx+S(n+1)+N(n+1)\\ x_{n+1}=x_{n}+h\left(k_{1}+2k_{2}+2k_{3}+k_{4}\right)/6 \end{array}\right..$$

**Figure 2:**
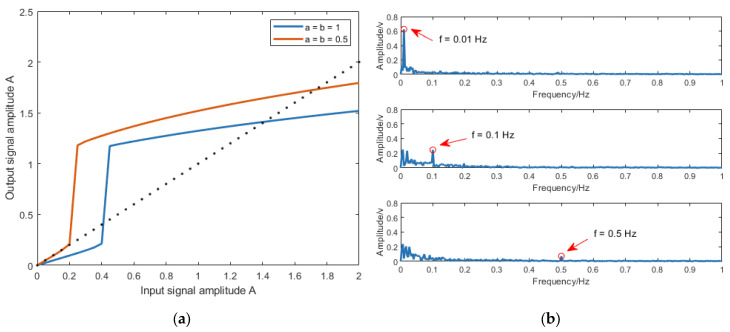
The characteristics of CBSR systems: (**a**) output saturation and (**b**) low-frequency selection.

To analyze the output saturation characteristic of the CBSR system, the parameter configurations are set as follows: the input signal is $S\left(t\right)=Asin(0.02\pi t)$, where A is bounded within the interval [0, 2]; $N\left(t\right)=0$; and the sampling frequency is 5 Hz. Figure 2a shows the output response results of the CBSR system.

In Figure 2a, as the amplitude of the input signal increases from zero, a sudden transition occurs in the output signal amplitude. The corresponding input amplitude at this critical point is defined as the threshold, which physically represents the potential barrier height of the SR system. When the input signal amplitude exceeds this threshold, the system’s output amplitude surpasses that of the input. However, further increases in input amplitude produce only marginal gains in output signal amplitude, ultimately yielding an output amplitude inferior to the input, thereby demonstrating output saturation behavior.

Additionally, to analyze the low-frequency selection characteristic of the CBSR system, the parameter configurations are set as follows: the input signal is $S\left(t\right)=0.5sin(2\pi ft)$, where f={0.01 Hz,0.1 Hz,0.5 Hz}; the sampling frequency is 5 Hz; the noise intensity D=0.5; and the potential function parameters are a=b=1. The output signal spectra of the CBSR coefficients under three distinct input frequencies are presented in Figure 2b.

In Figure 2b, a distinct amplification effect is observed exclusively for the 0.01 Hz input frequency, manifested as a dominant spectral peak in the output. In contrast, input signals at 0.1 Hz and 0.5 Hz exhibit amplitude attenuation, with their corresponding power spectra showing significant low-frequency noise enhancement and the appearance of multiple stochastic spectral peaks.

This phenomenon can be attributed to the fundamental operating principle of SR—the nonlinear system preferentially transfers noise energy to lower frequency bands while simultaneously facilitating resonant coupling between the noise components and periodic signals matching the system’s characteristic frequency. When processing input signals containing such matching frequency components, the system demonstrates effective signal amplification. Conversely, for non-resonant input frequencies, the system primarily outputs processed noise, which exhibits a decaying spectral profile originating from the low-frequency band.

Furthermore, the CBSR potential function incorporates second-order and fourth-order terms, whose higher-order nonlinearity inevitably increases computational complexity. This characteristic may consequently constrain its applicability in MMW fuze systems that demand stringent real-time processing capabilities.

## 3. The Fixed-Distance Target Detection Algorithm Based on Segmented Low-Order Bistable Stochastic Resonance

### 3.1. The Segmented Low-Order Bistable Stochastic Resonance

**Figure 3:**
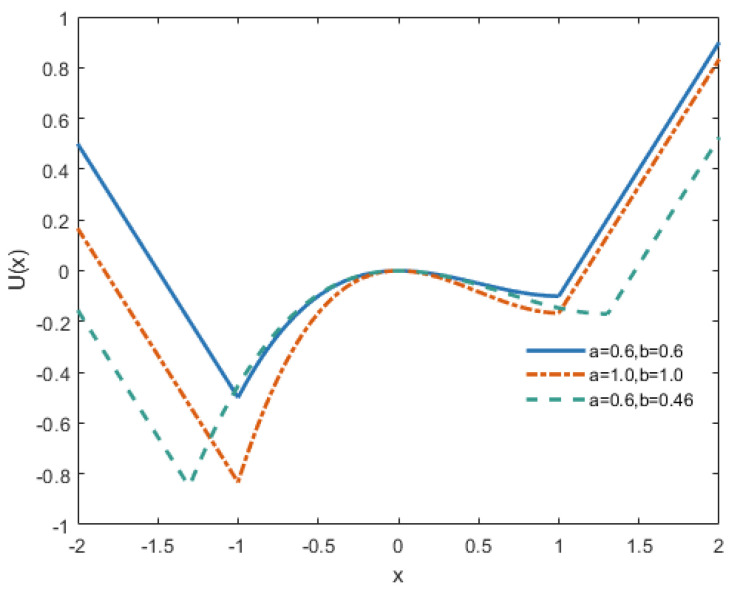
Curves of the potential model.

To overcome output saturation while reducing computational demands, this study proposes an SLOBSR model, which reconstructs the potential well section using second-order and third-order terms. The proposed potential function is as follows:

(11) $$U(x)=\left\{\begin{array}{l} -k\left(x+\frac{a}{b}\right)-\frac{5a^{3}}{6b^{2}},\;\;x<-a/b\\ -\frac{a}{2}x^{2}+\frac{b}{3}x^{3},-a/b\leq x\leq a/b\\ k\left(x-\frac{a}{b}\right)-\frac{a^{3}}{6b^{2}},\;\;x>a/b \end{array}\right.,$$

where potential function parameters a>0, b>0, and k>0. Figure 3 illustrates the potential function shape of the SLOBSR model, where k=1. The potential well geometry, including both depth and width characteristics, can be precisely controlled by parameters a and b. The potential function comprises two asymmetric potential wells with distinct depths, characterizing the SLOBSR as an asymmetric unsaturated bistable SR model.

The first derivative result of the potential function is as follows:

(12) $$\frac{dU(x)}{d(x)}=\left\{\begin{array}{l} -k,\;x<-a/b\\ -ax+bx^{2},-a/b\leq x\leq a/b\\ k,\;\;x>a/b \end{array}\right..$$

**Figure 4:**
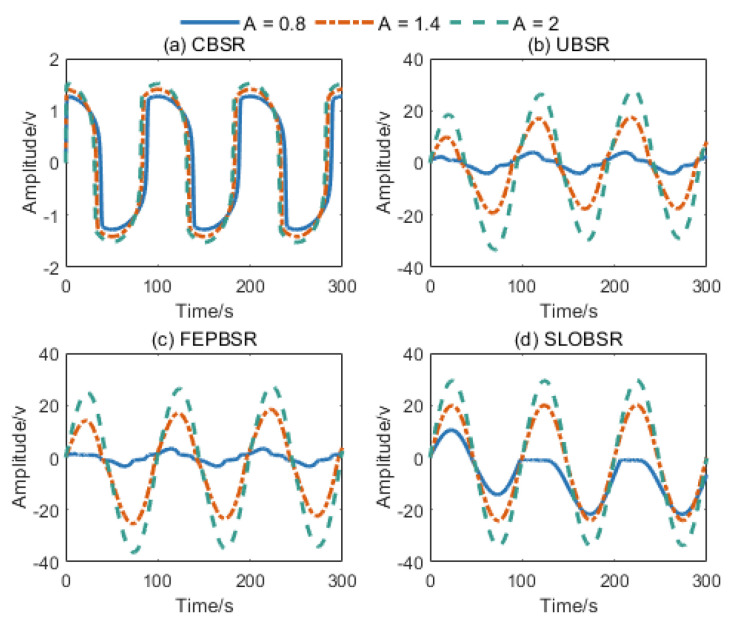
Output signals of (**a**) CBSR system, (**b**) UBSR system, (**c**) FEPBSR system, and (**d**) SLOBSR system.

To analyze the characteristics of the proposed approach, the simulation signal is utilized for researching the system response of the CBSR, UBSR, FEPBSR, and SLOBSR methods. The simulated signal is $S\left(t\right)=Asin(0.02\pi t)$, where A={0.8, 1.4, 2} and $N\left(t\right)=0$. The parameters of the potential function were configured as follows: a=b=1 for CBSR, a=b=1 for UBSR, a=b=1 and u=1.2 for FEPBSR [27], and a=b=0.5 and k=0.1 for SLOBSR. The parameters for CBSR, UBSR, and FEPBSR systems were selected from standard configurations, whereas the SLOBSR parameters were empirically chosen to demonstrate the inherent saturation characteristics of different SR methods. Figure 4 shows the output response results of each SR method. Notably, compared to CBSR, the UBSR, FEPBSR, and SLOBSR systems successfully overcame the output saturation limitation and generated significantly higher output signal amplitudes.

To analyze the output SNR of the SLOBSR system, the derivation commences from the Kramers rate formula, ultimately yielding the system’s output SNR expression. According to [32], the Kramers rate is computed as follows:

(13) $$R^{-1}=\frac{1}{D}\int_{-\infty }^{A}e^{-\frac{U(x)}{D}}\;dx\int_{-\infty }^{A}e^{\frac{U(x)}{D}}\;dx.$$

Therefore, the Kramers rate of the SLOBSR system can be obtained by substituting Equation (11) into Equation (13) as follows:

(14) $$\begin{array}{c} R_{-}^{-1}=\frac{1}{D}\int_{-\frac{a}{b}-\frac{5a^{3}}{6kb^{2}}}^{-\frac{a}{b}}e^{-\frac{1}{D}[-k\left(x+\frac{a}{b}\right)-\frac{5a^{3}}{6b^{2}}]}\;dx\int_{-\frac{a}{b}}^{0}e^{\frac{1}{D}(-\frac{a}{2}x^{2}+\frac{b}{3}x^{3})}\;dx\\ R_{+}^{-1}=\frac{1}{D}\int_{0}^{\frac{a}{b}}e^{-\frac{1}{D}(-\frac{a}{2}x^{2}+\frac{b}{3}x^{3})}\;dx\int_{\frac{a}{b}}^{\frac{a}{b}+\frac{a^{3}}{6kb^{2}}}e^{\frac{1}{D}[k\left(x-\frac{a}{b}\right)-\frac{a^{3}}{6b^{2}}]}\;dx \end{array}.$$

Using the Taylor expansion and considering only the constant term, Equation (14) can be calculated as follows:

(15) $$\begin{array}{c} R_{-}^{-1}=\frac{kb}{a}e^{-\frac{5a^{3}}{6Db^{2}}}\\ R_{+}^{-1}=\frac{kb}{a}e^{-\frac{a^{3}}{6Db^{2}}} \end{array}.$$

Following the theoretical derivation of asymmetric function SNR presented in [28], a deterministic relationship emerges between the SNR and Kramers rate, which is given by the following expression:

(16) $$\mathrm{SNR}=\frac{\pi {\left(Ax_{m}\right)}^{2}(R_{-}+R_{+})}{4D^{2}\left[1-\frac{1}{2}{\left(\frac{Ax_{m}}{D}\right)}^{2}\frac{{\left(R_{-}+R_{+}\right)}^{2}}{{\left(R_{-}+R_{+}\right)}^{2}+\omega_{0}^{2}}\right]}.$$

The SNR approximation is obtained by substituting Equation (15) into Equation (16) and neglecting the higher-order terms in the denominator,

(17) $$SNR\approx \frac{\pi A^{2}ka}{4D^{2}b}(e^{-\frac{5a^{3}}{6Db^{2}}}+e^{-\frac{a^{3}}{6Db^{2}}}).$$

**Figure 5:**
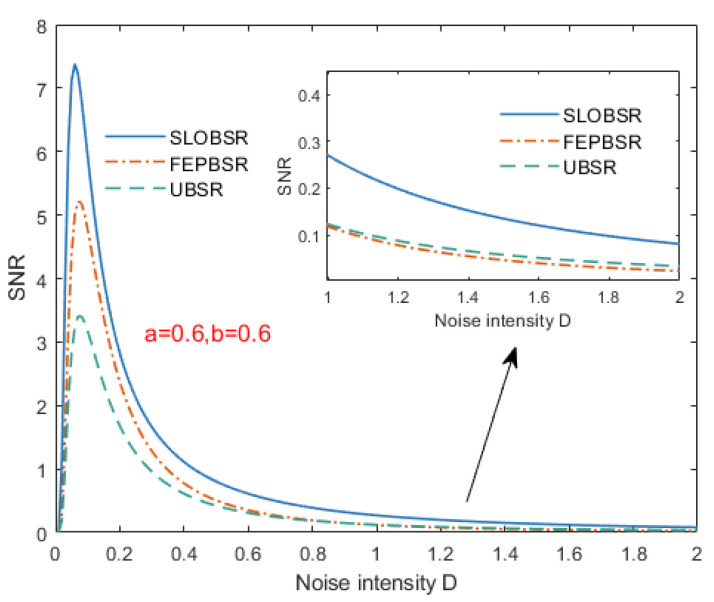
Theoretical output SNR of systems.

To comparatively analyze the theoretical SNR variation characteristics of the SLOBSR model, the SNR fluctuation curve versus noise intensity was plotted, where other parameters were fixed as follows: u=1.1 for FEPBSR and k=1 for SLOBSR. The results are presented in Figure 5.

As shown in Figure 5, the SLOBSR system exhibits a characteristic SNR profile where (1) SNR initially ascends with increasing noise intensity, peaking at optimal signal–noise synchronization, and (2) subsequent noise amplification causes progressive SNR attenuation. Compared to both the FEPBSR and UBSR systems, the SLOBSR method demonstrates superior theoretical output SNR. Notably, while FEPBSR exhibits higher SNR than UBSR under low-noise conditions, its performance advantage diminishes and eventually reverses as noise intensity increases.

### 3.2. The Fixed-Distance Target Detection Algorithm

To leverage the frequency-selective amplification properties of SR systems, a fixed-distance target detection algorithm is developed. When targets enter the fuze’s proximity, their echo signals contain characteristic periodic components. Subsequent nonlinear processing yields significantly enhanced SNR, enabling effective noise suppression in rainy environments.

#### 3.2.1. Input Signals Pre-Processing

For optimal SR operation, the input signal must satisfy the following two fundamental requirements: (1) contain periodic components and (2) maintain sufficiently low frequency characteristics. The constraints ensure optimal resonance performance, with detailed specifications provided below [21,33].

(18) $$\left\{\begin{array}{l} f\ll 1\\ f\leq f_{s}\cdot 1/50 \end{array}\right.,$$

where f is the signal frequency of the periodic signal S(t), and $f_{s}$ is the sampling frequency. To ensure compatibility with SR system requirements in Equation (18), the input signal typically requires preprocessing through the following two key operations: (1) single side-band amplitude modulation (SSB-AM) and (2) scale transformation (ST) [33]. The resulting modulated signal via SSB-AM can be expressed as follows:

(19) $$\left\{\begin{array}{l} S_{sample}\left(t\right)=Acos[2\pi {(f+f}_{c})t]+n_{1}(t)\\ S_{c}\left(t\right)=\cos\left(2\pi f_{c}t\right)\\ S_{m}\left(t\right)=S_{sample}\left(t\right)\times S_{c}\left(t\right)-H\left[S_{sample}\left(t\right)\right]\times H\left[S_{c}\left(t\right)\right]=Acos\left(2\pi ft\right)+n_{2}(t) \end{array}\right.,$$

where $S_{sample}\left(t\right)$ is the analog-to-digital sampled signal; $S_{c}(t)$ is the carrier signal; $S_{m}(t)$ is the modulated signal; and H[∗] signifies the Hilbert transform operation. $n_{1}\left(t\right)$ represents the noise component in the input sampled signal, while $n_{2}\left(t\right)$ denotes the SSB-AM modulated version of this noise component. The ST is quantified by the re-scaling ratio $R_{s}$, defined as the quotient of the fuze sampling frequency $F_{s}$ to the resampling frequency $F_{rs}$, expressed as follows:

(20) $$R_{s}=F_{s}/F_{rs}.$$

To address signal amplitude variability, amplitude normalization must be applied to the modulated signals. Resonance phenomena in SR systems require the periodic signal amplitude A to exceed the system’s potential barrier height. Consequently, conventional unity normalization is unsuitable under low SNR conditions. Through empirical investigation, this study establishes a normalization factor of 15 to ensure reliable SR operation. The sampled signal $S_{sample}\left(t\right)$ undergoes SSB-AM, ST, and amplitude normalization to generate the input signal S(t)+N(t) in Equation (8) for the SR system. Then, the output signal is obtained by randomly initializing x while adaptively adjusting the potential function parameters of the SR system.

#### 3.2.2. Parameter Optimization for SR System

In practical applications, the noise intensity in input signals remains fixed, precluding direct noise adjustment to achieve resonance matching. Consequently, resonance optimization must be accomplished through parametric adaptation of the system’s potential function.

To identify the optimal parameter combination, this study employs PSO due to its derivative-free nature, minimal parameter requirements, straightforward implementation, rapid convergence properties, and robust global search capabilities [34]. The PSO implementation begins with randomized initialization of particle positions encoding SR system parameters, which are evaluated through SR processing to generate output signals. The algorithm iteratively refines these positions by achieving the following: (1) adjusting inertia weights and learning factors to balance global exploration and local exploitation; (2) elitist selection preserving top-performing particles to prevent premature convergence; and (3) velocity clamping and boundary handling to ensure feasible SR parameter ranges. Convergence is achieved when maximum iterations are reached. For computational efficiency, the PSO algorithm implementation utilizes a population size of 30 particles with 10 iterations. Furthermore, the SNR serves as the optimization metric, calculated for experimental signals as follows:

(21) $$\left\{\begin{array}{l} \mathrm{SNR}=10{log}_{10}[P_{s}/(P_{n}-P_{s})]\\ P_{s}=\sum_{i=g-h}^{g+h}|X(i){|}^{2}\\ P_{n}=\sum_{i=1}^{N/2}|X(i){|}^{2} \end{array}\right.,$$

where the signal power $P_{s}$ and noise power $P_{n}$ are calculated from the measured signal spectrum X. *g* represents the spectral line index closest to the target frequency. To account for the picket fence effect, the target signal region is defined as ±*h* bins centered at *g*, with *h* set to 3 in this implementation. N denotes the length of the input signal.

#### 3.2.3. The Target Decision Method

When processing echo signals of MMW fixed-distance fuze under rainy conditions, three distinct scenarios may occur, namely: extra-range target (beyond system range), off-range target (within range but at non-specified distances), and On-range target (at precisely specified distances). Similarly, there are three scenarios for the input and output signals of an SR system; they are as follows:

1. Extra-Range Target:
  Input: Pure noise (no target signature);
  Output: Low-frequency noise accumulation;
  Spectral signature: Multiple peaks of noise in the power spectrum.
2. Off-Range Target:
  Input: Noise and off-range periodic components;
  Output: Low-frequency noise accumulation with original frequency components;
  Spectral signature: Multiple peaks of noise and off-range periodic components.
3. On-Range Targets:
  Input: Noise and on-range periodic components;
  Output: Amplified target signal with suppressed noise;
  Spectral signature: Dominant unimodal peak at target frequency.

The SR system demonstrates distinct output characteristics for the three aforementioned scenarios. Crucially, resonance phenomena between noise and signal components only occur in Case 3. Therefore, the specified target periodic signal exhibits substantial SNR improvement, and a pronounced unimodal distribution can be observed in the spectral domain. In contrast, Cases 1 and 2 exhibit multiple spectral peaks due to low-frequency noise accumulation. This fundamental difference enables effective discrimination of Case 3 through kurtosis analysis [35], thereby facilitating MMW fuze distance determination under rainy conditions. The kurtosis is computed as follows:

(22) $$\mathrm{Kur}=\frac{\frac{1}{N}\sum_{i=1}^{N}{\left(p_{i}-\bar{p}\right)}^{4}}{{[\frac{1}{N}\sum_{i=1}^{N}{\left(p_{i}-\bar{p}\right)}^{2}]}^{2}},$$

where $p_{i}$ is the power spectrum of the output signal obtained by calculating the squared magnitude of the output signal’s Fourier spectrum. $\bar{p}$ is the average value of the power spectrum $p_{i}$.

### 3.3. The Main Process of the Proposed Method

The main processing steps of the method proposed in this study are as follows:

1. First, sampling of MMW fuze echo beat signals under rainy conditions.
2. The sampled signals undergo pre-processing, including SSB-AM, ST, and amplitude normalization. The frequency of carrier signals in SSB-AM corresponds to the predetermined distance.
3. The pre-processed signals are fed into the SLOBSR system, with system parameters (a, b, k) optimized via the PSO algorithm.
4. Calculate the power spectral kurtosis of the SLOBSR system output signal.
5. Threshold detection is performed on the spectral kurtosis. If the kurtosis exceeds the predetermined threshold, the fixed-distance function of the MMW fuze is accomplished; otherwise, the aforementioned procedure is repeated iteratively.

**Figure 6:**
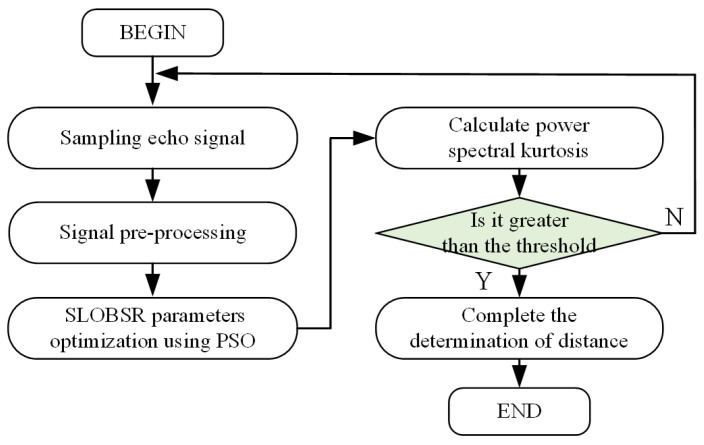
Main flow of the method proposed in this study.

The main processing steps of the method proposed are shown in Figure 6.

## 4. Simulation Results

This section presents a performance evaluation of the fixed-distance algorithm based on the SLOBSR method through numerical simulations. Computational experiments were conducted on a workstation equipped with an AMD Ryzen 5 4600H CPU and 16 GB DDR4 RAM, utilizing MATLAB R2018b. The main simulation parameters are shown in Table 1.

Under this parameter configuration, the target echo signal frequency at 9 m is $f_{b}=4∆F/(Tc)\cdot R=3.6\;MHz$. The target echo signal undergoes SSB-AM with a 3.5 MHz carrier frequency, resulting in a modulated output signal at 0.1 MHz. Following ST, the resampled signal with a $F_{sr}=F_{s}/R_{s}=5\;Hz$ sampling frequency and $0.1\;MHz/R_{s}=0.01\;Hz$ characteristic frequency was obtained and subsequently employed as the input to the SR system.

### 4.1. The Performance of the Fixed-Distance Algorithm Based on the SLOBSR Method

**Figure 7:**
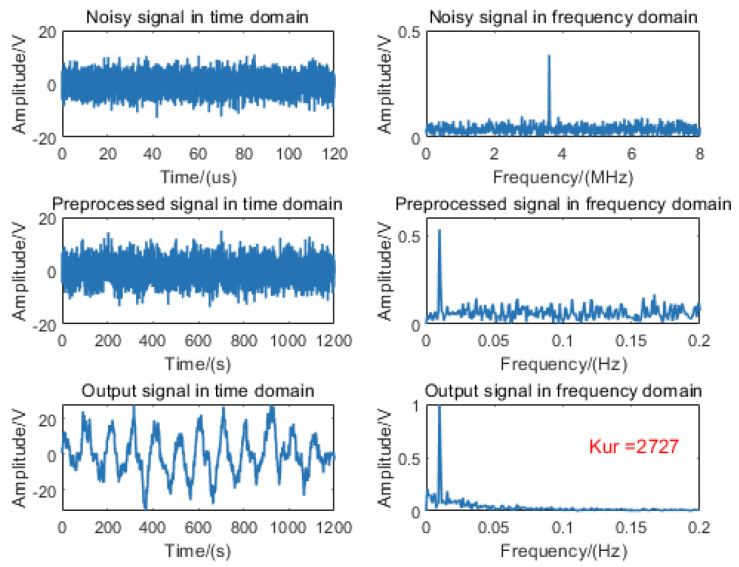
Processing diagram of the fixed-distance algorithm for a 9 m target signal at −10 dB SNR condition.

Based on the MMW fuze echo signal model under rainy conditions, echo signals were simulated through parametric variation of both target distance and rainfall intensity. For algorithm validation, a beat signal with a 9 m target and −10 dB SNR was selected as the input for the fixed-distance algorithm, with the processing results presented in Figure 7.

The signal processing results demonstrate that the fixed-distance algorithm successfully induces nonlinear SR in the echo signal, facilitating efficient noise-to-signal energy conversion. Spectral analysis reveals significant noise suppression and prominent enhancement of the target frequency component, exhibiting excellent unimodal characteristics with a kurtosis value of 2727.

**Figure 8:**
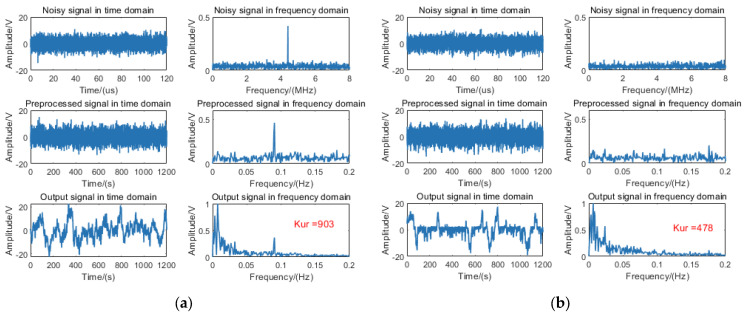
Processing diagram of the fixed-distance algorithm for (**a**) an 11 m target signal at −10 dB SNR condition and (**b**) a pure noise signal.

Similarly, two distinct input signals were selected for the fixed-distance algorithm: (1) a beat signal with an 11 m target range and −10 dB SNR, and (2) pure noise without target signal components. The corresponding processing results are presented in Figure 8a and Figure 8b, respectively.

The spectral analysis in Figure 8a reveals that the output signal maintains the characteristic frequency component corresponding to the 11 m target. However, low-frequency noise accumulation generates multiple spectral peaks in the lower frequency band, indicating the absence of resonance effects between the target signal and noise components. In Figure 8b, the output signal spectrum similarly exhibits low-frequency noise-induced spurious peaks, with no discernible resonance phenomena. The measured kurtosis values for these two conditions were 903 and 478, respectively, neither of which exceeded the predefined threshold.

**Figure 9:**
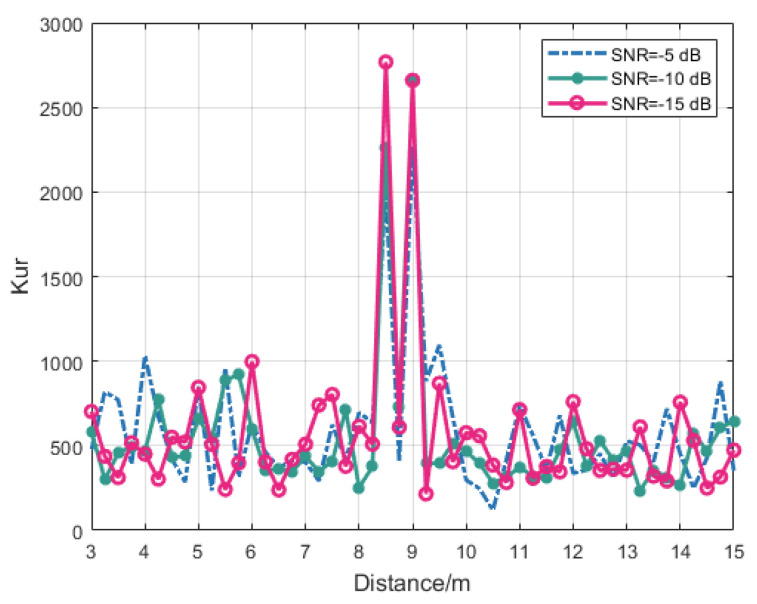
The kurtosis characteristics of the fixed-distance algorithm output signals across varying target distances.

The simulation results conclusively demonstrate that kurtosis threshold detection enables reliable identification of specified-distance target signals at −10 dB SNR conditions. The signal normalization procedure in the fixed-distance algorithm yields consistent output for pure noise inputs without target signals. Subsequent analysis evaluates the kurtosis characteristics of processed echo signals across varying target distances (3–15 m) at SNRs of −5 dB, −10 dB, and −15 dB. The kurtosis values of the output signals processed by the fixed-distance algorithm were systematically analyzed, as illustrated in Figure 9.

Spectral analysis reveals that output signal kurtosis exceeds the threshold (≥2000) exclusively at target distances of 8.5 m and 9 m. Notably, the 8.5 m target generates a signal frequency slightly below the carrier frequency. Due to the frequency symmetry inherent in cosine modulation, the SSB-AM produces a noise-corrupted signal containing low-frequency periodic components, thereby meeting the input requirements for SR. This phenomenon leads to elevated kurtosis values at 8.5 m, effectively establishing a secondary detection mechanism that enhances the ranging algorithm’s robustness. For all other target distances, the processed output signals exhibited kurtosis below the threshold, typically remaining under 1000. The simulation results demonstrate that the proposed SLOBSR-based fixed-distance algorithm achieves reliable target distance determination for echo signals with SNRs ranging from −15 dB to −5 dB.

### 4.2. Comparison with Other Methods

To validate the performance of the proposed method, three commonly used noise reduction techniques in fuzes were selected for comparison: EMD, VMD, and AF. Higher SNR of single-frequency signals leads to more pronounced unimodal characteristics and consequently higher kurtosis values in the fixed-distance algorithm outputs. Based on this property, a comparative analysis of SNR enhancement performance among EMD, VMD, and least mean squares-based AF (LMS-AF) was conducted. The VMD implementation utilized two intrinsic mode functions, while both EMD and VMD selected the decomposed component with the maximum Pearson correlation coefficient as the denoised output. The LMS-AF was configured with a 16-tap filter, using the pure single-frequency signal corresponding to the 9 m target as the desired signal. All parameters were optimized through a comprehensive evaluation of signal length, spectral composition, noise suppression capability, and computational efficiency to ensure a balanced comparison.

To comparatively evaluate the SNR enhancement performance of the proposed SLOBAR algorithm against other SR methods, systematic comparisons with UBSR, FEPBSR, and AHBSR approaches were conducted. All SR methods utilized identical SSB-AM, ST, amplitude normalization, and PSO procedures to ensure experimental consistency. It should be noted that the re-scaling ratio $R_{s}$ of AHBSR was specifically configured as 1.5 MHz to meet its unique low-frequency periodic signal processing requirements. The evaluation employed the 9 m target signal as input under three SNR conditions (−5 dB, −10 dB, and −15 dB), with the corresponding output SNR performance metrics systematically quantified in Table 2.

The comparative results demonstrate that SR methods consistently achieve superior output SNR performance compared to EMD, VMD, and LMS-AF. Among SR approaches, FEPBSR exhibits marginally lower SNR enhancement than UBSR, attributable to its reduced efficacy under high-noise conditions. The AHBSR method demonstrates superior output SNR than UBSR at −5 dB but shows reduced performance at −10 dB and −15 dB. Most significantly, the proposed SLOBAR method achieves the highest output SNR across all three noise conditions, demonstrating consistent superiority over all benchmarked methods.

It should be noted that to reduce computational overhead, this study employed relatively small population sizes and limited iteration counts during PSO-based parameter optimization. Consequently, none of the stochastic resonance (SR) models may have achieved their theoretically optimal SNR outputs. The comparative analysis was, therefore, conducted based on multiple simulation trials under these constrained optimization conditions. The results specifically indicate that the proposed SLOBSR method achieves superior SNR performance compared to benchmark techniques when optimization cycles are restricted.

To quantitatively assess the computational complexity of the proposed methodology, the execution time for all algorithms was measured, with the average processing time required for 100 independent runs presented in Table 3. It should be emphasized that all SR methods utilized identical SSB-AM, ST, amplitude normalization, and PSO procedures, and all comparison methods involve the entire process from obtaining the input signal to outputting the final signal.

The computational complexity analysis reveals that EMD and LMS-AF exhibit shorter execution times, but in practical implementations they demonstrate limited SNR enhancement capability and desired signal setting challenges, respectively. Among SR methods, FEPBSR requires the longest processing time due to multiple square root operations, followed by AHBSR with its exponential computation requirements. In contrast, both UBSR (0.07 s) and SLOBSR (0.05 s) achieve superior computational efficiency through low-order polynomial operations. Notably, SLOBSR demonstrates the lowest computational complexity among all SR methods, attributable to its optimized low-order implementation.

## 5. Experimental Results

To further validate the efficacy of the proposed methodology, experimental verification was conducted.

### 5.1. Description of Simulated Rainfall Scenario

**Figure 10:**
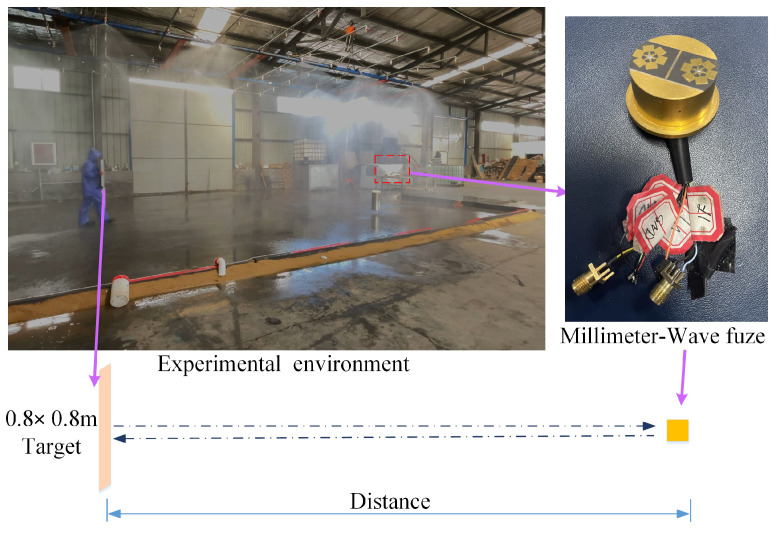
The experimental rainfall scenario.

The experimental scenario is shown in Figure 10. A simulated rainfall environment was established within a controlled chamber measuring 13 m (length) × 8 m (width) × 5 m (height). Multiple rainfall nozzles were installed on the ceiling to generate controlled rainfall, with rainfall intensity regulated through hydraulic pressure adjustment. The MMW fuze (specifications detailed in Table 1) was positioned within the test chamber, while a 0.8 m × 0.8 m metal plate target was placed at 9 m and 11 m distances for static testing.

Statistical analysis of the experimental data was conducted to determine the SNR range of the echo signals, with quantitative results systematically presented in Table 4. Experimental results demonstrate that the echo signal of MMW fuzes experiences significant SNR degradation under rainy conditions, with a maximum observed reduction of 5.57 dB. This substantial SNR deterioration adversely impacts the precise ranging capability of MMW fuzes in rainy environments.

### 5.2. Validation of the Proposed Method on Measured Data

**Figure 11:**
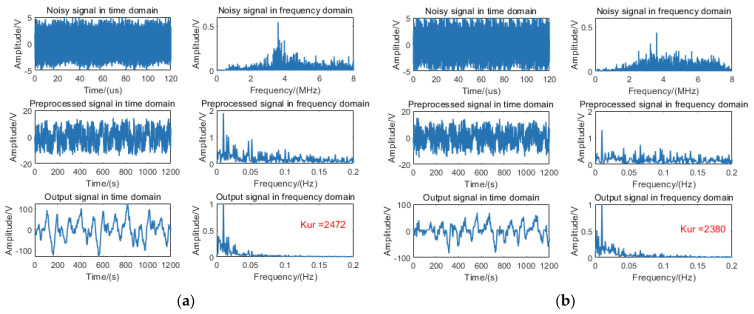
Processing diagram of the fixed-distance algorithm for a 9 m target signal: (**a**) without rain and (**b**) with rain.

To validate the efficacy of the proposed algorithm on measured data, the 9 m target signal was processed using the fixed-distance algorithm. The algorithmic outputs without rain and with rain are, respectively, illustrated in Figure 11a and Figure 11b.

Empirical measurements reveal that echo signals with rain exhibit significantly elevated noise floor levels compared to those without rain. Subsequent processing via the fixed-distance algorithm induces SR phenomena in both signal types, manifesting as pronounced unimodal spectral distributions with kurtosis values surpassing the predefined detection threshold.

**Figure 12:**
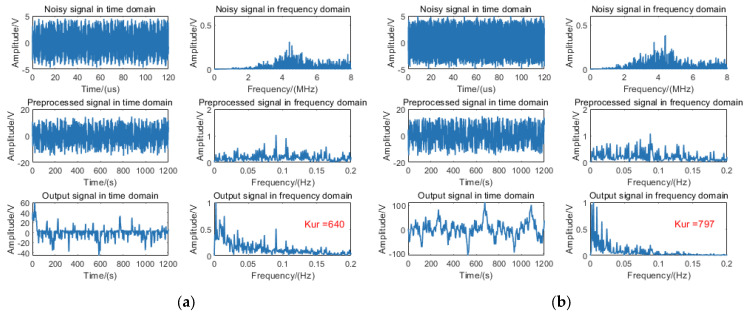
Processing diagram of the fixed-distance algorithm for an 11 m target signal: (**a**) without rain and (**b**) with rain.

Furthermore, the 11 m target signal was processed using the fixed-distance algorithm; the algorithmic outputs without rain and with rain are, respectively, illustrated in Figure 12a and Figure 12b.

Experimental data analysis indicates that while the 11 m target signal is detectable, it fails to meet the necessary conditions for SR. Consequently, the fixed-distance algorithm processing does not yield a resonance effect, resulting in kurtosis values below the threshold.

**Figure 13:**
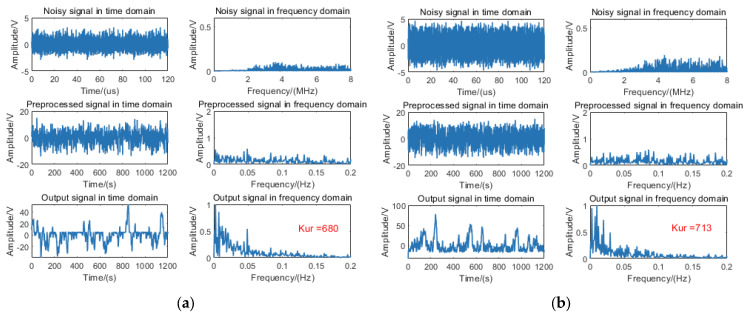
Processing diagram of the fixed-distance algorithm for a no-target signal: (**a**) without rain and (**b**) with rain.

Additionally, the no-target signal was processed using the fixed-distance algorithm; the algorithmic outputs without rain and with rain are, respectively, illustrated in Figure 13a and Figure 13b.

Experimental analysis demonstrates that when processing pure noise signals without target signatures using the fixed-distance algorithm, noise energy accumulation occurs in the low-frequency domain of the frequency spectrum, resulting in kurtosis values below threshold.

To validate the fixed-distance accuracy of the proposed method for measurement data, kurtosis values were systematically computed under various experimental conditions, with quantitative results tabulated in Table 5.

Rainfall induces a significant increase in the noise floor of the received signals, consequently reducing the lower bound of the kurtosis value distribution. Notably, experimental results with and without rain demonstrate that the output kurtosis values exceed the detection threshold only during processing of the 9 m target signal, thereby confirming the effectiveness of the proposed method.

### 5.3. Comparison with Other Methods on Measured Data

To comparatively evaluate the SNR enhancement performance of the proposed SLOBAR algorithm against other methods, the SNR enhancement performance of various methodologies was quantitatively evaluated using measured data, with comparative results presented in Table 6.

The results demonstrate that all methods achieved measurable SNR enhancement under the experimental conditions. Compared to no-rain conditions, conventional methods (EMD, VMD, and LMS-AF) exhibit marginal SNR improvement (about 0.3 dB) under rainy conditions, whereas SR methods demonstrate approximately 1 dB enhancement, confirming superior noise energy utilization efficiency of SR methods. Among SR methods, FEPBSR shows inferior performance. While AHBSR outperforms UBSR without rain, this advantage reverses with rain, aligning with simulation results. Notably, the proposed SLOBSR obtains superior SNR enhancement across all environmental conditions, achieving a remarkable 9.94 dB SNR improvement under rainy conditions.

## 6. Conclusions

To address the critical challenge of SNR degradation in MMW fuze echo signals under rainy conditions while satisfying real-time processing constraints, this study proposes a novel fixed-distance target detection algorithm based on the SLOBSR system. The effectiveness and superiority of the fixed-distance algorithm have been rigorously validated through both simulations and experimental studies, yielding three principal findings:

First, the developed segmented low-order potential function successfully reduces computational complexity while maintaining nonlinear enhancement capabilities. The constructed SLOBSR system demonstrates superior performance through comprehensive saturation characteristic analysis and theoretical SNR output verification.

Second, the proposed detection algorithm integrates the following three key innovations: (i) advanced signal pre-processing incorporating SSB-AM, ST, and amplitude normalization; (ii) intelligent parameter optimization via PSO; and (iii) robust distance determination through kurtosis-based threshold selection. Experimental validation confirms reliable target identification capability for echo signals with SNRs between −15 dB and −5 dB.

Third, comparative performance analysis reveals that the algorithm exhibits the highest SNR enhancement and minimal computational overhead compared to FEPBSR, UBSR, and AHBSR methods across both simulated and experimental datasets, achieving 9.94 dB average SNR improvement in measured rainfall data and significantly outperforming conventional methods including EMD, VMD, and LMS-AF.

This study successfully applies the stochastic resonance method to MMW fuze fixed-distance detection, introducing a groundbreaking approach for low-SNR target detection. The proposed algorithm achieves significant SNR enhancement under rainy conditions while satisfying stringent real-time processing requirements, enhancing anti-rainfall interference robustness.

## Figures and Tables

**Table 1:** Simulation parameters.

Parameter	Values
Operating frequency $f_{0}$	35 GHz
Modulation method	Triangular wave
Modulation period T	20 us
Modulation bandwidth ∆F	600 MHz
Fuze sampling frequency $F_{s}$	50 MHz
Carrier signal frequency $f_{c}$	3.5 MHz
Re-scaling ratio $R_{s}$	10 MHz
Signal length N	6144
Kurtosis threshold	1500

**Table 2:** Output signals SNR across different methodologies.

Input SNR	EMD	VMD	LMS-AF	FEPBSR	UBSR	AHBSR	SLOBSR
−5 dB	0.1019	5.6917	6.6558	6.4188	7.2145	7.3471	7.7342
−10 dB	−4.3579	1.3705	1.7365	2.8175	4.5501	4.0347	4.7520
−15 dB	−8.5302	−3.0654	−4.4165	0.6309	1.5887	−0.1801	1.6322

**Table 3:** Running time of different methods.

EMD	VMD	LMS-AF	FEPBSR	UBSR	AHBSR	SLOBSR
0.0175	0.6766	0.0015	1.2470	0.0688	0.1484	0.0515

**Table 4:** Range of measured data SNR.

	Without Rain	With Rain
9 m	[−8.9410, −6.5711]	[−14.5141, −8.3234]

**Table 5:** The kurtosis range under different experimental conditions.

	With No Target	With 11 m Target	With 9 m Target
Without rain	[329, 859]	[255, 777]	[1744, 2523]
With rain	[240, 1138]	[178, 1088]	[1641, 2512]

**Table 6:** Average SNR enhancement performance of various methodologies.

	EMD	VMD	LMS-AF	FEPBSR	UBSR	AHBSR	SLOBSR
With rain	1.2791	3.2208	4.1467	7.9017	8.4762	8.6733	8.7650
Without rain	1.6316	3.4524	4.4266	9.2002	9.6782	9.5213	9.9352

## Data Availability

The data are available from the corresponding author upon reasonable request.

## Abbreviations

The following abbreviations are used in this manuscript:

MMW	millimeter-wave
SNR	signal-to-noise ratio
SLOBSR	segmented low-order bistable stochastic resonance
PSO	particle swarm optimization
RCS	radar cross-section
EMD	Empirical Mode Decomposition
VMD	Variational Mode Decomposition
AF	adaptive filtering
SR	stochastic resonance
CBSR	classical bistable stochastic resonance
UBSR	unsaturated bistable stochastic resonance
FEPBSR	fractional exponential power bistable stochastic resonance
AHBSR	asymmetric hybrid bistable stochastic resonance
SSB-AM	single side-band amplitude modulation
ST	scale transformation
LMS-AF	least mean squares adaptive filtering

## References

[1] Cui Z., Song S., Xu L. (2009). Principle of Proximity Fuze.

[2] Chen S., Liu M., Lu F., Xing M. (2019). A Target Identification Method for the Millimeter Wave Seeker via Correlation Matching and Beam Pointing. Sensors.

[3] Budalal A.A.H., Islam M.R., Abdullah K., Rahman T.A. (2020). Modification of Distance Factor in Rain Attenuation Prediction for Short-Range Millimeter-Wave Links. IEEE Antennas Wirel. Propag. Lett..

[4] Varotsos G.K., Aidinis K., Nistazakis H.E. (2022). Average BER Performance Estimation of Relayed THz Links with Losses, Molecular Attenuation, Adverse Weather Conditions, Turbulence and Generalized Pointing Errors. Photonics.

[5] Ishimaru A. (1997). Wave Propagation and Scattering in Random Media.

[6] Zang S., Ding M., Smith D., Tyler P., Rakotoarivelo T., Kaafar M.A. (2019). The Impact of Adverse Weather Conditions on Autonomous Vehicles: How Rain, Snow, Fog, and Hail Affect the Performance of a Self-Driving Car. IEEE Veh. Technol. Mag..

[7] Yang R., Ma H., Li L., Zhu C. Research on the influence of rainfall on millimeter wave Doppler fuze. Proceedings of the 2011 Second International Conference on Mechanic Automation and Control Engineering.

[8] Hasirlioglu S., Riener A. (2020). A General Approach for Simulating Rain Effects on Sensor Data in Real and Virtual Environments. IEEE Trans. Intell. Veh..

[9] Steinhauser D., Held P., Thöresz B., Brandmeier T. Towards Safe Autonomous Driving: Challenges of Pedestrian Detection in Rain with Automotive Radar. Proceedings of the 2020 17th European Radar Conference (EuRAD).

[10] Kawaguchi T., Shinotsuka K., Malterer S. Experimental Verification of Rainfall Impact on Sparse Array Radar. Proceedings of the 2024 IEEE Radar Conference (RadarConf24).

[11] Gourova R., Krasnov O., Yarovoy A. Analysis of rain clutter detections in commercial 77 GHz automotive radar. Proceedings of the 2017 European Radar Conference (EURAD).

[12] Zhan C., Zhang S., Sun C., Chen S. (2024). Anti-Rain Clutter Interference Method for Millimeter-Wave Radar Based on Convolutional Neural Network. Remote Sens..

[13] Wang J., Hu P., Su X., Han Y. (2014). Application of EMD Denoising Method in Signal Processing of FMCW Radar. Sci. Technol. Eng..

[14] Zhou W., Hao X., Yang J., Duan L., Yang Q., Wang J. (2023). Interference Mitigation Method for Millimeter-Wave Frequency-Modulation Continuous-Wave Radar Based on Outlier Detection and Variational Modal Decomposition. Remote Sens..

[15] Pang Z., Song C., Liu B. (2024). Separation of Noisy Multitone Signals Based on Variational Mode Decomposition. J. Appl. Phys..

[16] Lu C., Li G., Xiong B. (2010). Anti-Noise for LFM Fuze Based on Variable Step Size LMS Algorithm. J. Nav. Aeronaut. Astronaut. Univ..

[17] An G., Tong Q., Zhang Y., Liu R., Li W., Cao J., Lin Y. (2021). An Improved Variational Mode Decomposition and Its Application on Fault Feature Extraction of Rolling Element Bearing. Energies.

[18] Haykin S. (1996). Adaptive Filter Theory.

[19] Sun C., Shrivastava A., Singh S. Revisiting Unreasonable Effectiveness of Data in Deep Learning Era. Proceedings of the 16th IEEE International Conference on Computer Vision (ICCV).

[20] Cui H., Guan Y., Chen H., Deng W. (2021). A Novel Advancing Signal Processing Method Based on Coupled Multi-Stable Stochastic Resonance for Fault Detection. Appl. Sci..

[21] Yang Z., Li Z., Zhou F., Ma Y., Yan B. (2022). Weak Fault Feature Extraction Method Based on Improved Stochastic Resonance. Sensors.

[22] Benzi R., Sutera A., Vulpiani A. (1981). The mechanism of stochastic resonance. J. Phys. A Math. Gen..

[23] Wang C., Qiao Z., Huang Z., Xu J., Fang S., Zhang C., Liu J., Zhu R., Lai Z. (2022). Research on a Bearing Fault Enhancement Diagnosis Method with Convolutional Neural Network Based on Adaptive Stochastic Resonance. Sensors.

[24] Cui L., Xu W. (2022). A New Piecewise Nonlinear Asymmetry Bistable Stochastic Resonance Model for Weak Fault Extraction. Machines.

[25] Rousseau D., Varela J.R., Chapeau-Blondeau F. (2003). Stochastic resonance for nonlinear sensors with saturation. Phys. Rev. E Stat. Nonlinear Soft Matter Phys..

[26] Qiao Z., Lei Y., Lin J., Jia F. (2017). An adaptive unsaturated bistable stochastic resonance method and its application in mechanical fault diagnosis. Mech. Syst. Signal Process..

[27] Chen J., Zhang X., Chen Z., Zi Y., Chen Y., Shi Z. (2024). A Fractional Exponential Power Bistable Stochastic Resonance Method for Rolling Bearing Weak Features Extraction. IEEE Trans. Instrum. Meas..

[28] Liu G., Chu X. (2025). An OFDM Signal Enhancement and Demodulation Method Based on Segmented Asymmetric Bistable Stochastic Resonance. IEEE Access.

[29] Wang S., Yuan Y., Zhang M. (2024). Study on Weak Signal Feature Extraction Based on Asymmetric Hybrid Bistable Stochastic Resonance. IEEE Trans. Instrum. Meas..

[30] Wiscombe W.J. (1980). Improved Mie scattering algorithms. Appl. Opt..

[31] Leng Y., Wang T., Guo Y., Wu Z. (2007). Study of the property of the parameters of bistable stochastic resonance. Acta Phys. Sin..

[32] Zhao W., Wang J., Wang L. (2013). The unsaturated bistable stochastic resonance system. Chaos Interdiscip. J. Nonlinear Sci..

[33] Tan J., Chen X., Wang J., Chen H., Cao H., Zi Y., He Z. (2009). Study of frequency-shifted and re-scaling stochastic resonance and its application to fault diagnosis. Mech. Syst. Signal Process..

[34] Kennedy J., Eberhart R. Particle swarm optimization. Proceedings of the ICNN’95—International Conference on Neural Networks.

[35] Wang J., He Q., Kong F. (2015). Adaptive Multiscale Noise Tuning Stochastic Resonance for Health Diagnosis of Rolling Element Bearings. IEEE Trans. Instrum. Meas..

